# Electrochemical Evaluations of Fractal Microelectrodes for Energy Efficient Neurostimulation

**DOI:** 10.1038/s41598-018-22545-w

**Published:** 2018-03-12

**Authors:** Hyunsu Park, Pavel Takmakov, Hyowon Lee

**Affiliations:** 1 Weldon School of Biomedical Engineering, Birck Nanotechnology Center, Center for Implantable Devices, Purdue University, West Lafayette, IN USA; 2 Division of Biology, Chemistry and Materials Science; Office of Science and Engineering Laboratories, Center for Devices and Radiological Health, U.S. Food and Drug Administration, White Oak Federal Research Center, Silver Spring, MD USA

## Abstract

Advancements in microfabrication has enabled manufacturing of microscopic neurostimulation electrodes with smaller footprint than ever possible. The smaller electrodes can potentially reduce tissue damage and allow better spatial resolution for neural stimulation. Although electrodes of any shape can easily be fabricated, substantial effort have been focused on identification and characterization of new materials and surface morphology for efficient charge injection, while maintaining simple circular or rectangular Euclidean electrode geometries. In this work we provide a systematic electrochemical evaluation of charge injection capacities of serpentine and fractal-shaped platinum microelectrodes and compare their performance with traditional circular microelectrodes. Our findings indicate that the increase in electrode perimeter leads to an increase in maximum charge injection capacity. Furthermore, we found that the electrode geometry can have even more significant impact on electrode performance than having a larger perimeter for a given surface area. The fractal-shaped microelectrodes, despite having smaller perimeter than other designs, demonstrated superior charge injection capacity. Our results suggest that electrode design can significantly affect both Faradaic and non-Faradaic electrochemical processes, which may be optimized to enable a more energy efficient design for neurostimulation.

## Introduction

Electrical stimulation of the nervous system is used ubiquitously to replace and restore lost bodily functions in patients with a number of neurological impairments including neuromotor deficit^1^, vision and hearing loss^2,3^, chronic pain^4^, and epilepsy^5^. In 2015, the total market size for various implantable neural stimulation devices that target spinal cord, cochlear, cerebral cortex, and other peripheral nerves (e.g., Sacral, Vagus nerve), exceeded $4.9 billion with the annual growth rate of 17%^6^. The increasing popularity for neurostimulation has fueled the demand for more precise targeting of neural substrates. For example, vision prostheses now feature more than 1000 stimulating microelectrodes with a diameter of 100 *μ*m, and manufacturers of cortical stimulation devices have begun to create higher density electrodes for stimulating various deep brain structures^7^. The advances in microfabrication technologies has made it possible for researchers to investigate feasibility of high density microscale electrode arrays even with more complex geometries^8–12^. While it is relatively easy to design, and manufacture smaller stimulating electrodes, doing so can functionally limit the amount of electrical charge that can be delivered through smaller surface area.

Moreover, the reduction in electrode size also increases the overall electrical load of battery-powered stimulation systems. A conventional implantable neural stimulation system, which consists of three major components: implantable pulse generator (IPG), electrical leads, and neurointerfacing electrodes, have average lifetime of 4–6 years^13^. Although the lifetime of these chronically implanted systems differ widely depending on individualized stimulation parameters and usage, minimizing the electrical load is imperative for ensuring long-term utility of these systems^14,15^. Therefore, enhancing the resolution of stimulation using smaller microscale electrodes requires a careful consideration on overall impact of electrode design in terms of stimulation performance as well as its impact on device longevity.

There are significant efforts in the field to increase the efficiency of neurostimulators by decreasing microelectrode impedance or increasing the charge transfer capability. For instance, electrode material and the surface morphology were found to have significant impact on electrochemical impedance and charge transfer capacity. Iridium oxide (IrOx) electrodes have been studied widely to show superior charge injection capability than Pt-based electrodes^16,17^. Similarly, researchers have also touted poly(3,4-ethylenedioxythiophene) (PEDOT) for having a higher charge injection limit than Pt and IrOx electrodes^18^. However, PEDOT has been reported to shows mechanical failure such as delamination and cracking during chronic stimulation^19^. Despite many groups working to develop higher performing microelectrodes using various electrode materials, platinum (Pt) remains as the gold-standard for commercial neurostimulation devices, especially for cortical stimulation.

In terms of morphology, researchers have explored various fabrication methods to improve performance of electrodes. De Haro *et al*., demonstrated that electroplated Pt has lower impedance and higher corrosion resistance than sputtered Pt, which can improve the lifetime of the microelectrode^20^. Sputtered material such as IrOx and titanium nitride have been demonstrated to be superior than evaporated IrOx due to difference in nanoscale surface morphology^16,17^. Shota *et al*., showed that microelectrode composed of IrOx and Pt-black with nanoscale roughness has a lower impedance and high charge-injection capability than flat microelectrode^21^. Boehler *et al*., reported that Pt microelectrode with nanograss structure has reduced impedance and strong adhesion to metallized substrate^22^.

The impact of electrochemical performance on electrodes with high perimeter-to-surface area (PSA) has also been well documented^23–25^. Electrochemical impedance spectroscopy (EIS) from circular microscale electrodes with different diameters showed that smaller microelectrode corresponded to a higher impedance, which can be attributed to an increase in the solution resistance and shorter charging time of capacitive double-layer on electrode^26^. Grill *et al*., reported that conventional deep brain stimulation electrodes split into smaller segments with higher PSA ratios have higher stimulation efficiency and smaller energy load for IPGs despite showing no significant differences in impedance between single electrode and segmented ones^27^. Cogan *et al*. confirmed that increasing the PSA ratio of the IrOx microelectrode lowered the electrode impedance and improved the charge injection limit perhaps due to reduction in access resistance and increasing ion flux to the electrode surface^28^. These reports suggest that increasing the PSA may be an effective way of improve electrode performance.

One easy way to achieve high PSA in a small footprint is to use fractals. For this reason, fractal designs have been used widely in antennae designs to achieve multi-band capability and to reduce size^29–32^. Recently, several groups have begun to explore the utility of fractal designs in neurostimulation as well. Golestanirad *et al*. reported a numerical model of modified Sierpinski carpet electrode that requires 22% less energy to activate a given population of neurons^33^. Most recently, another group reported similar results using a numerical model of an electrode with branching fractal design that can penetrate deeper into the neural substrate compared to Euclidean electrodes, suggesting better neurostimulation performance^34^. Compared to conventional Euclidean electrode geometry, which results in a significant current density gradient across the electrode surface^35–37^, fractal electrodes are able to deliver a more uniformly high current density across the surface^34^. However, all of the published work to date focused on numerical analyses of various fractal designs without systematic electrochemical evaluations. Two important questions remain to be explored: (1) do fractal electrodes exhibit similar charge injection limit improvement as shown in high PSA electrodes? and (2) by what mechanism?

In this work, we electrochemically examined the role of electrode geometry in terms of PSA ratio and shape using custom microfabricated electrode arrays. Four types of electrodes with identical surface area but varying PSA ratios were created: circular, fractal, serpentine I, and serpentine II (Table 1). The circular electrodes with 100 *μ*m diameter was chosen as a representative Euclidean design with the lowest PSA ratio. The fractal and serpentine I electrodes were designed to have the same PSA ratio to explore whether different shapes would have an impact on electrochemical performance of microelectrodes. Serpentine II electrode had the highest PSA ratio. Serpentine designs were used for their space-filling capacity and potential utility in flexible bioelectronics^38–40^. Based on prior literature, we expected to show increased electrode performance with increasing PSA regardless of the electrode shape.

**Table 1:** Dimensions of the microelectrodes. Measured dimensions were taken from representative samples.

		Circle	Fractal	Serpentine I	Serpentine II
As Designed	Perimeter [mm]	0.3142	1.998	1.998	3.156
	Area [mm^2^]	7.854 × 10^−3^	7.854 × 10^−3^	7.854 × 10^−3^	7.854 × 10^−3^
	Perimeter/Area [mm^−1^]	40	254	254	400
	Footprint [L × W]	D = 100 *μ*m	157 × 157 *μ*m^2^	94 × 148 *μ*m^2^	126 × 133 *μ*m^2^
Measured	Perimeter [mm]	0.3016	1.886	1.912	3.078
	Area [mm ^2^]	7.238 × 10^−3^	7.214 × 10^−3^	7.357 × 10^−3^	7.573 × 10^−3^
	Perimeter/Area [mm^−1^]	42	261	259	406
	Footprint [L × W]	D = 96 *μ*m	153 × 153 *μ*m^2^	93 × 149 *μ*m_2_	126 × 130 *μ*m^2^

Using numerical modeling, we quantified the total charge injected and the current density around the microelectrodes. We evaluated the electrochemical performance of each electrodes using cyclic voltammetry, EIS, and voltage transient analysis. The cathodal and the total charge storage capacity of each electrode were calculated from the time integral of the current in cyclic voltammogram, which is related to the charge injection capability of electrode. The EIS measurements were performed in phosphate buffered saline to characterize capacitive and non-Faradaic processes. Additionally, we used ferri-ferrocyanide red-ox pair as a probe to characterize mass-transport to the electrodes associated with Faradaic processes that occur during charge injection with electrical stimulation^41^. To investigate the effects of the geometry on charge injection limit, we compared the maximum negative potential excursion and the maximum driving voltage of the different shaped microelectrodes using voltage transient analysis with different charge injection levels. Finally, the energy consumption from different microelectrodes was quantified from the cathodic potential transient and the applied current waveform.

Our results indicate that the electrode shape may play a more significant role in charge injection capability of microelectrodes than previously reported. We found that fractal microelectrodes exhibit markedly superior electrode performance than other PSA-matched microelectrodes. Here we provide empirical evidence to suggest that the improved microelectrode performance in non-Euclidean electrodes may be due to lower access resistance and more efficient mass-transport in high PSA and fractal electrodes. Furthermore, these results suggest the possibility of further design optimization towards more energy efficient stimulating electrodes that may enable more reliable chronic electrical neuromodulation.

## Results

**Figure 1:**
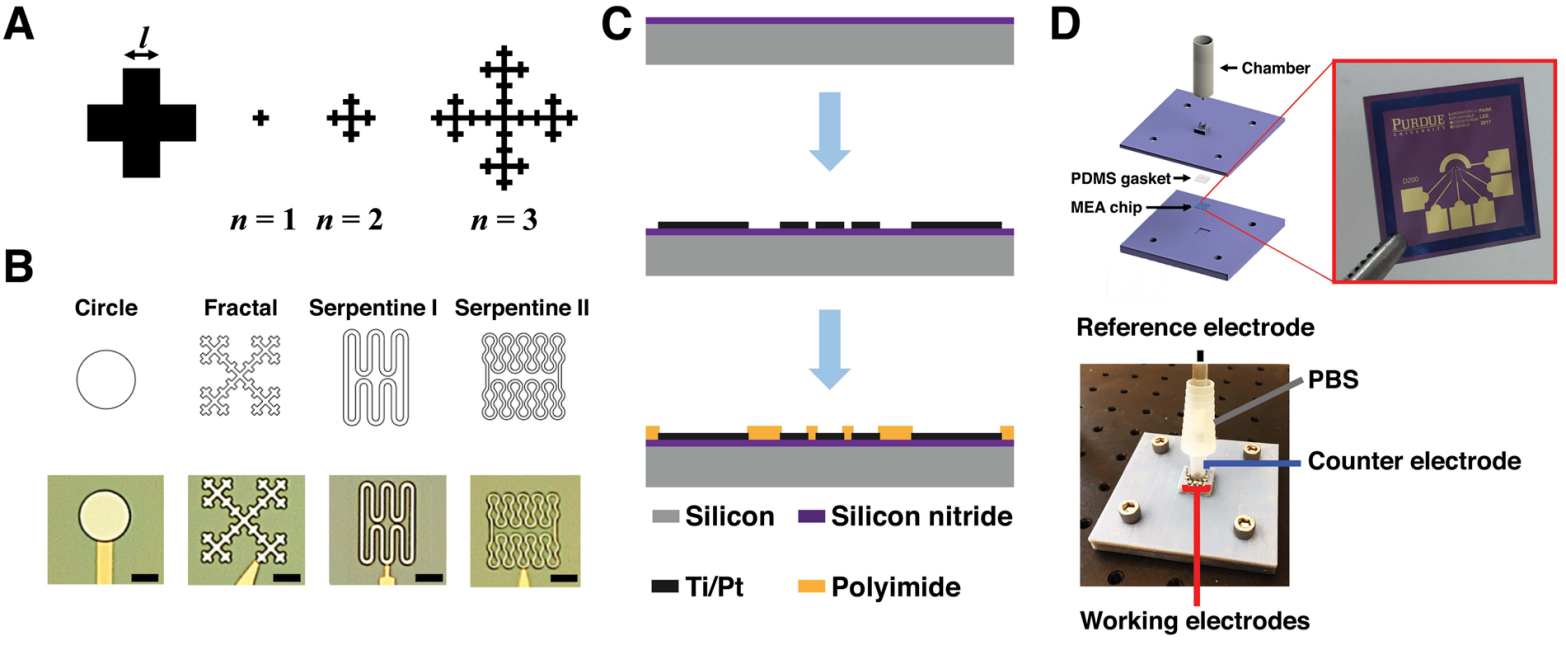
(**A**) Viseck fractal design parameters at different iteration levels with the minimum feature size *l*. (**B**) Planar geometry with different shapes and optical micrographs of the fabricated microelectrodes. Sale bars = 50 *μ*m. (**C**) Fabrication sequence of microelectrode arrays: deposition of 500 nm silicon nitride: evaporation of Ti/Pt; and polyimide passivation layer coating followed by RIE for opening. (**D**) Experimental setup with 3D printed fixture to accommodate the microelectrode arrays for electrochemical measurements.

The geometry of fabricated microelectrodes in Fig. 1. Table 1 summarizes the difference between the design and fabricated samples. In general, electrodes were smaller than the original design but PSA ratios remain close to the expected value. The SA for fractal electrodes were the smallest compared to serpentine II by approximately 5%.

### Current density distribution in 3D COMSOL model

The impact of constant voltage (−0.6 V) stimulation on the four electrode designs was examined using AC/DC module in 3D COMSOL model. The impedance of each electrode design was also modeled in COMSOL using 10 mV AC voltage perturbation from 10 Hz to 100 kHz. The electrochemical process including chemical reactions on the electrode surface and mass transfer kinetics were ignored in this simulation because the mathematical handling of electrochemical reactions from non-uniform current density along irregular geometry is difficult to model accurately^42^. Therefore, most modeling studies have focused on the simulation of the Faradaic process on the circular shaped microelectrodes with limited conditions such as just one red-ox pair with the parameters from simplified chemical reaction assumption^43,44^. However, the numerical simulation for non-Faradaic process without consideration of mass transfer kinetics are widely utilized because it can still provide insight on solution resistance, charge transfer resistance, double layer capacitance and electrical stimulation efficiency for various electrode geometries^27,45^.

**Figure 2:**
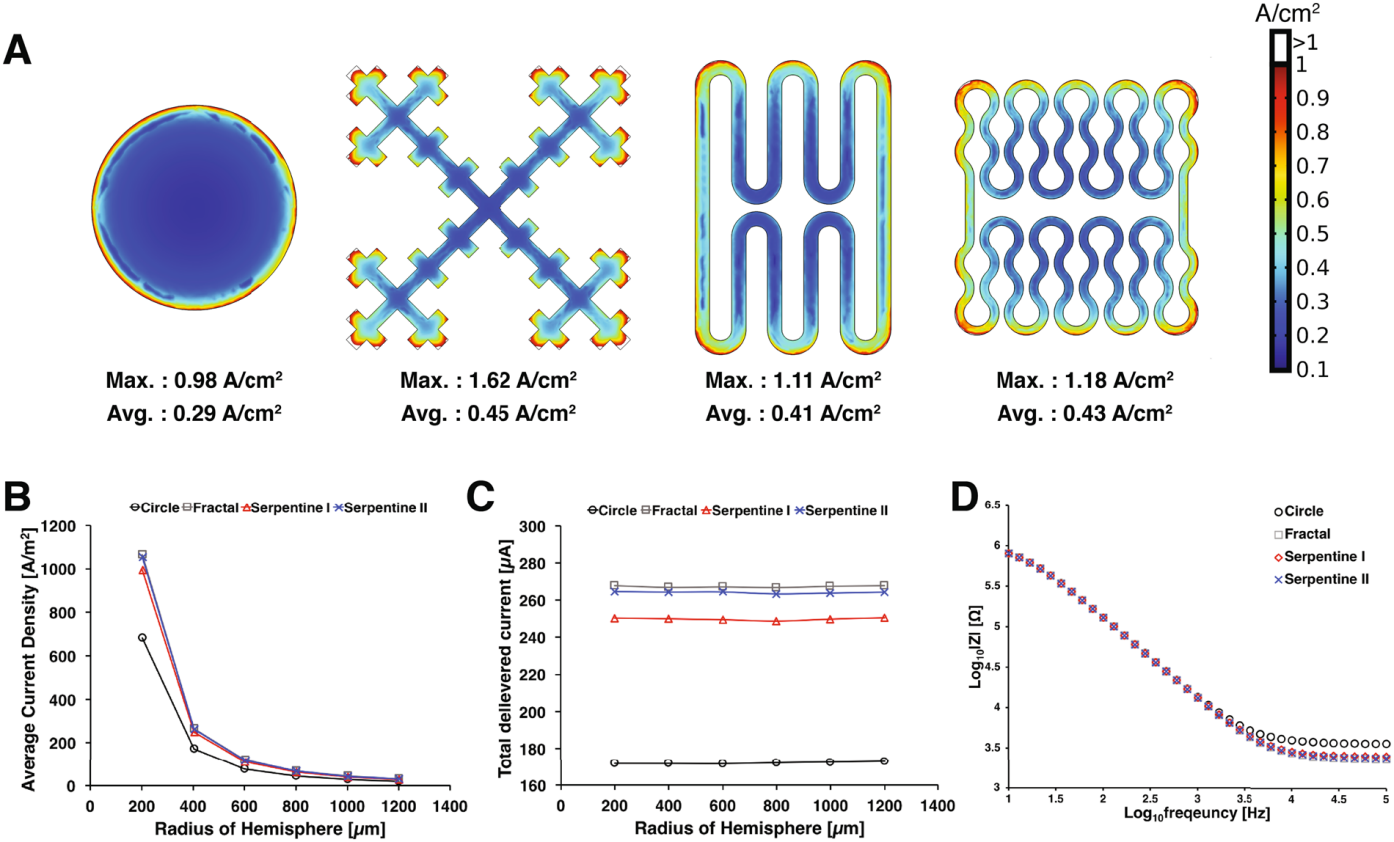
The current density distribution of different electrode design using the voltage-control stimulation (−0.6 V). (**A**) The current density surface plot for each microelectrode design. Both the maximum and the average current density across the electrode surface was highest for the fractal design followed by serpentine II, serpentine I, and circular electrode. (**B**) Average current density as a function of distance away from each electrode center. (**C**) The total delivered current on hemispheric boundaries. (**D**) Simulated impedance Bode plot of each microelectrode design.

Figure 2A shows that the fractal design produces the highest current density around the microelectrode, followed by other shapes in the order of perimeter-to-area ratio. The average current density followed the same trend with the fractal electrode having highest value at all distances away from the electrode surface. The fractal electrode was able to inject the highest amount of current of 267 *μ*A compared to 172 *μ*A for the circular electrode (i.e., 55% increase) ((Fig. 2B). The serpentine II and serpentine I electrodes delivered the current of 264 *μ*A and 250 *μ*A, respectively (Fig. 2C).

Figure 2D shows the impedance calculated for each microelectrode design. Typically, the impedance at high frequency (>10 kHz) corresponds to the solution resistance while the impedance at low frequency are affected by the charge transfer resistance and the diffusion-limited Faradaic processes. In our simulation, the fractal design had the lowest impedance at 100 kHz, followed by serpentine II, serpentine I, and the circular design. This result corresponds to the same trend found for the average current density (Fig. 2B) and the total delivered current (Fig. 2C), which suggests that lower solution resistance may be responsible for better charge injection capacity. At lower frequency (10 Hz), the impedance of each electrode design was not significantly distinguishable from each other, which reflects the lack of Faradaic components.

### Cyclic voltammetry and electrochemical impedance spectroscopy

**Figure 3:**
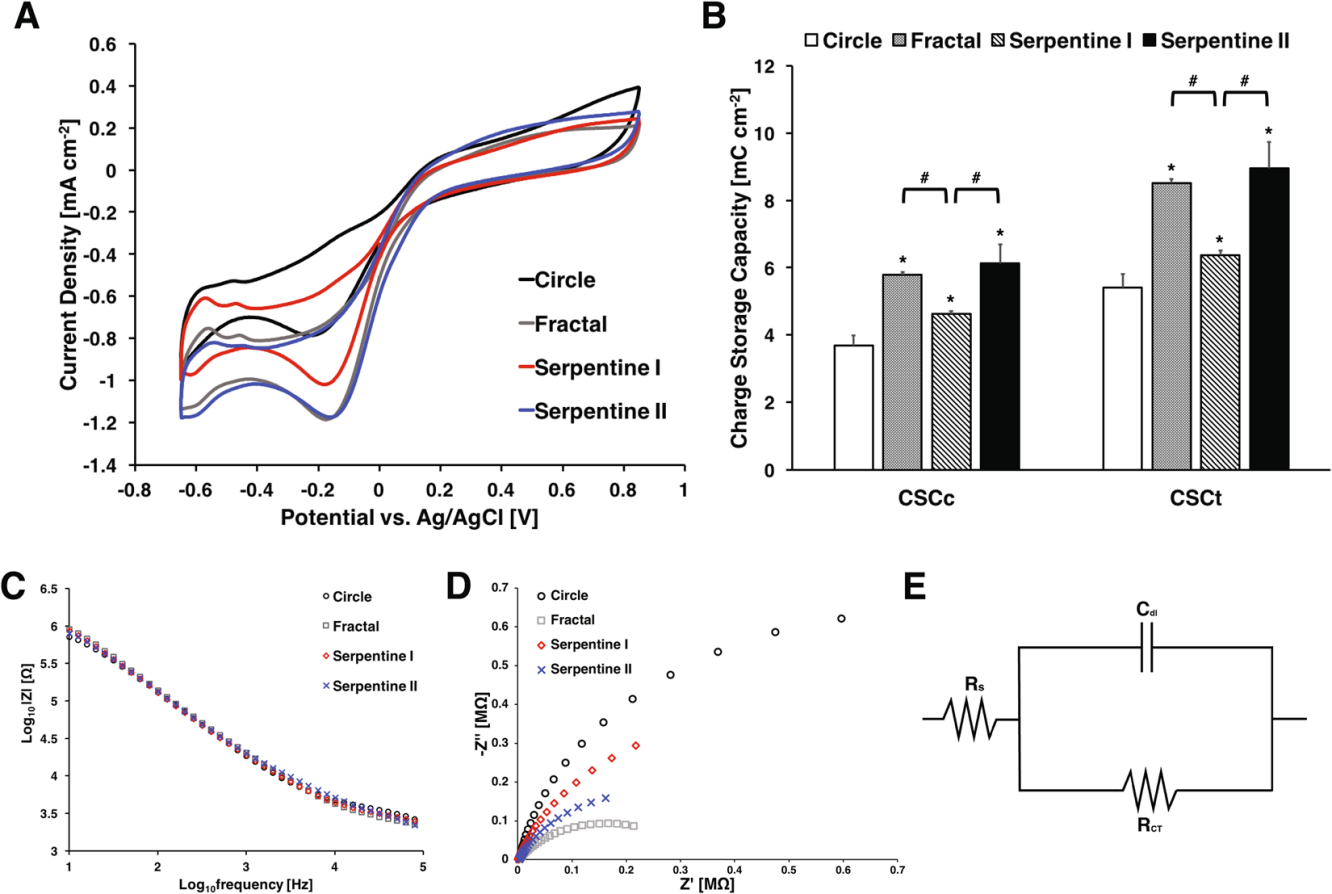
(**A**) Comparison of representative cyclic voltammogram of Pt microelectrodes in PBS. (**B**) Charge storage capacity of each microelectrode (n = 5 for each). ANOVA results revealed significant differences (p < 0.01) as compared to circular electrodes (*), and significant differences (p < 0.01) between fractal and serpentine I, serpentine I and serpentine II. (**C**) Representative impedance spectra of microelectrodes with different shapes The impedance spectra of microelectrodes in PBS. (**D**) Nyquist plot of microelectrodes (**E**) Randle’s circuit for equivalent circuit model analysis.

Figure 3A shows CV responses of the microelectrodes with different shapes measured in PBS from −0.65 V to 0.85 V at a sweep rate of 50 mVs^−1^. The voltammographs show that the fractal, serpentine I, and serpentine II electrodes with higher perimeter-to-area ratio all have lower cathodic current density than the circular electrode. From the voltammographs, we calculated the total and cathodal charge storage capacities (CSC) of each electrodes using the following equation^16^:

1 $$CSC=\frac{1}{\nu A}{\int }_{{E}_{c}}^{{E}_{a}}|i|\,dE\,(C/c{m}^{2})$$

with the potential versus Ag/AgCl reference electrode *E*, the measured current *i*, the positive and negative potential range E_*a*_ and E_*c*_, the surface area of the microelectrode *A*, and the scan rate *ν*. For CSC_*c*_, only the cathodic current was used for calculation, and both anodic and cathodic currents were used for CSC_*t*_. The average CSC_*c*_ and CSC_*t*_ for each electrode design are shown in Table 2. The CSC_*c*_ and CSC_*t*_ of the electrodes were compared using one-way ANOVA with Tukey’s HSD post-hoc analyses. The results indicated that the CSC_*c*_ and CSC_*t*_ of circular microelectrode were significantly smaller than the other microelectrodes with higher perimeter-to-area ratio (p < 0.01) (Fig. 3B). In contrast with the FEM result, serpentine II electrodes had the highest CSC values rather than the fractal design although the difference between the two were not statistically significant. Interestingly, CSC values for fractal electrodes were significantly higher than serpentine I electrodes even though they share the same PSA and SA, which suggests that the electrode shape may have a significant impact on electrode performance.

**Table 2:** CSC_*c*_ and CSC_*t*_ of each microelectrode.

		Circle	Fractal	Serpentine I	Serpentine II
CSC [mC cm^−2^]	CSC_*c*_	3.69 ± 0.31	5.79 ± 0.10	4.62 ± 0.10	6.13 ± 0.55
	CSC_*t*_	5.42 ± 0.39	8.51 ± 0.13	6.37 ± 0.13	8.94 ± 0.79

Figure 3C shows representative impedance spectra of each microelectrode design in PBS. Although there were relatively small differences, the high PSA electrodes exhibited lower impedance at high frequency. At lower impedance, however, the circular microelectrode had the lowest impedance. The difference between simulated and measured impedance values are listed shown in Supplementary Fig. 1 and Supplementary Table 1.

Unlike the Bode plot, the Nyquist representation of EIS data showed significant differences between electrodes (Fig. 3D). Using Randle’s circuit as an equivalent model (Fig. 3E, the values of solution resistance R_*s*_, the charge transfer resistance R_*CT*_, and the double layer capacitance C_*dl*_ were estimated. The fractal electrode had the lowest R_*S*_ and R_*CT*_, and the highest C_*dl*_ (Table 3).

**Table 3:** Estimated parameters of equivalent circuit model for each electrode in PBS.

	R_*S*_ [Ω]	R_*CT*_ [Ω]	C_*dl*_ [F]
Circle	3050 ± 141.3	3.032 ± 0.203 × 10^6^	1.410 ± 0.209 × 10^−8^
Fractal	1066 ± 402.4	1.379 ± 0.944 × 10^6^	2.269 ± 0.162 × 10^−8^
Serpentine I	2304 ± 193.6	2.762 ± 0.473 × 10^6^	1.731 ± 0.156 × 10^−8^
Serpentine II	1609 ± 251.8	2.269 ± 0.218 × 10^6^	1.929 ± 0.073 × 10^−8^

**Figure 4:**
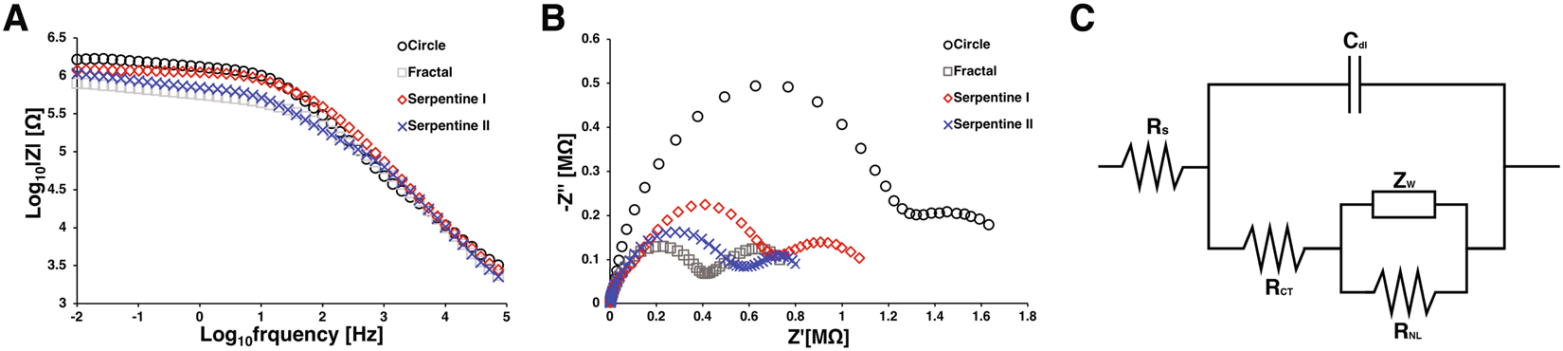
(**A**) The impedance spectra of microelectrodes in a 10 mM ferri-ferrocyanide + 0.1 M KCl solution. (**B**) Nyquist plot of microelectrodes in a 10 mM ferri-ferrocyanide + 0.1 M KCl solution. (**C**) Modified Randle’s circuit for equivalent circuit model analysis.

To distinguish the impact of mass transport on microelectrode performance, EIS was performed again in ferri-ferrocyanide, which is electrochemically reversible analyte with differing charges and relatively large ion size^41^. The Bode plot showed that each microelectrode has different impedance at low frequency range (<10 Hz) in which the impedance is dominated by mass transfer kinetics (Fig. 4A). Figure 4B shows the Nyquist plot with two identifiable semi-circles corresponding to different electrochemical processes. The diameter of the larger loop represents the charge transfer resistance whereas the smaller second loop indicates the impedance due to the diffusion of electroactive species to the electrode surface.

The Nyquist plots were fitted with an equivalent circuit model (Fig. 4C) to extract the values of R_*S*_, R_*CT*_, C_*dl*_, the Warburg coefficient W, and the nonlinear resistance related to hemi-spherical diffusion R_*NL*_^46^. The estimated values of each parameter from different electrode designs (n = 5 each) indicated that the fractal electrodes have the lowest R_*S*_, R_*CT*_, W, and R_*NL*_, and the highest C_*dl*_, which suggests that the fractal design has substantially lower overall resistance due to both Faradaic and non-Faradaic processes (Table 4 and Supplementary Fig. 2).

**Table 4:** Estimated parameters of equivalent circuit model for each electrode measured in 10 mM potassium ferri-ferrocyanide + 0.1 M KCl.

	R_*S*_ [Ω]	R _*CT*_ [Ω]	C_*dl*_ [F]	W [Ω*s*^−1/2^]	R _*NL*_ [Ω]
Circle	1666 ± 14	2.076 ± 0.017 × 10^6^	1.232 ± 0.805 × 10^−8^	3.459 ± 0.218 × 10^6^	10.040 ±1.431 × 10^5^
Fractal	475 ± 10	0.762 ± 0.147 × 10^6^	5.009 ± 1.147 × 10^−8^	0.131 ± 0.042 × 10^6^	4.672 ± 1.466 × 10^5^
Serpentine I	723 ± 64	1.367 ± 0.140 × 10^6^	2.940 ± 0.136 × 10^−8^	2.431 ± 0.173 × 10^6^	6.816 ± 0.029 × 10^5^
Serpentine II	550 ± 40	1.053 ± 0.551 × 10^6^	1.606 ± 0.101 × 10^−8^	1.532 ± 0.623 × 10^6^	5.675 ± 0.367 × 10^5^

### Voltage transients

**Figure 5:**
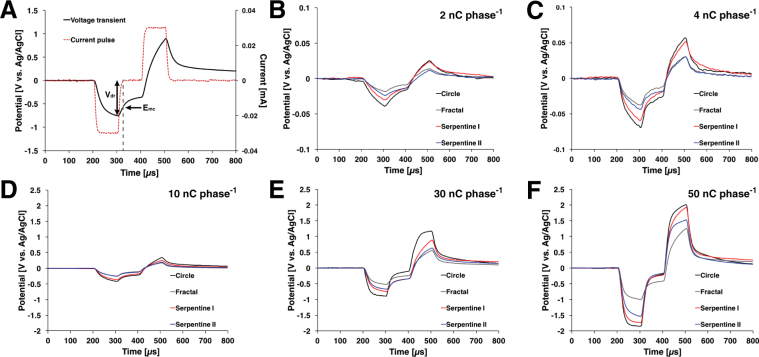
(**A**) Representative voltage transient of microelectrode with biphasic, symmetrical current pulse was applied at 50 Hz highlighted with maximum negative potential excursion (E_*mc*_) and maximum driving voltage (V_*dr*_). (**B**–**F**). Comparison of the voltage-transient responses of different microelectrodes at various total charge per phases (n = 5 for each). Overall, the fractal design had the lowest E_*mc*_ and V_*dr*_.

The voltage transient responses from the four electrodes (n = 5 each) were compared using constant current pulses at five different amplitudes (2 nC, 4 nC, 10 nC, 30 nC, and 50 nC per phase) at a frequency of 50 Hz (Fig. 5). The potential in the interphase region was 0 V versus Ag/AgCl sat. As can be seen in Fig. 5E, the fractal electrodes did not reach the −0.6 V water window limit until 30 nC/phase whereas all other electrodes exceed the water window by then.

**Figure 6:**
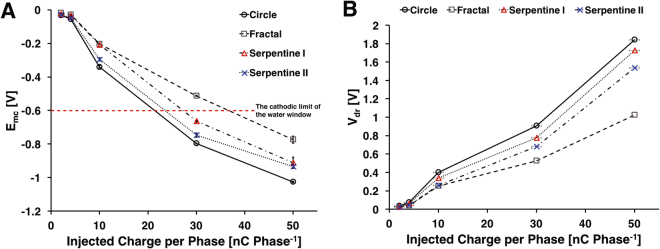
(**A**) Maximum negative potential excursion of the microelectrodes for each electrode design. Estimated charge injection limit (at −0.6 V) for each electrode: circle – 295.07 *μ*C/cm^2^ fractal – 510.507 *μ*C/cm^2^ serpentine I – 318.817 *μ*C/cm^2^ serpentine II – 359.527 *μ*C/cm^2^. Maximum driving voltage. Note that the fractal electrodes required significantly smaller amount of V_*dr*_ for any given injected charge levels.

The maximum negative potential E_*mc*_ is another way to estimate charge injection capacity of a stimulating electrodes. From the voltage transient response, E_*mc*_ can be estimated as the electrode potential at the end of cathodic current pulse^28^. A comparison of the E_*mc*_ for the four electrode designs is shown in Fig. 6A and Supplementary Fig. 3. In general, the fractal electrodes had the lowest E_*mc*_, followed by serpentine II, serpentine I, and the circular electrodes, which suggests highest charge injection capacity for the fractal design. Post-hoc pairwise comparison (p < 0.01) using Tukey’s test indicated that the fractal electrodes had statistically lower E_*mc*_ than all other electrodes at any charge injection level except against serpentine II at 10 nC/phase. Similarly, serpentine II electrodes were had statistically lower E_*mc*_ than serpentine I or circular electrodes except against serpentine I at 50 nC/phase. Serpentine I electrodes had statically lower E_*mc*_ than circular electrodes at all charge injection levels.

The maximum driving voltage (V_*dr*_) is the highest potential required to deliver the current pulse. The lower V_*dr*_ indicates that less energy is required to deliver a given charge to electrode surface. Figure 6B shows different V_*dr*_ required to deliver different charge levels to the four electrode designs under evaluation. One-way ANOVA with post-hoc pairwise comparison using Tukey’s test showed that the fractal electrode needed the lowest V_*dr*_ than any other electrodes at any charge injection levels. However, there was no significant difference between V_*dr*_ of serpentine I and circular electrode at 10 nC/phase. Similar to E_*mc*_ results, despite having the highest PSA ratio, the V_*dr*_ of serpentine II electrodes were significantly higher than fractal electrodes, which suggests that there may be additional geometric effects that influence electrode performance.

### Energy consumption

**Figure 7:**
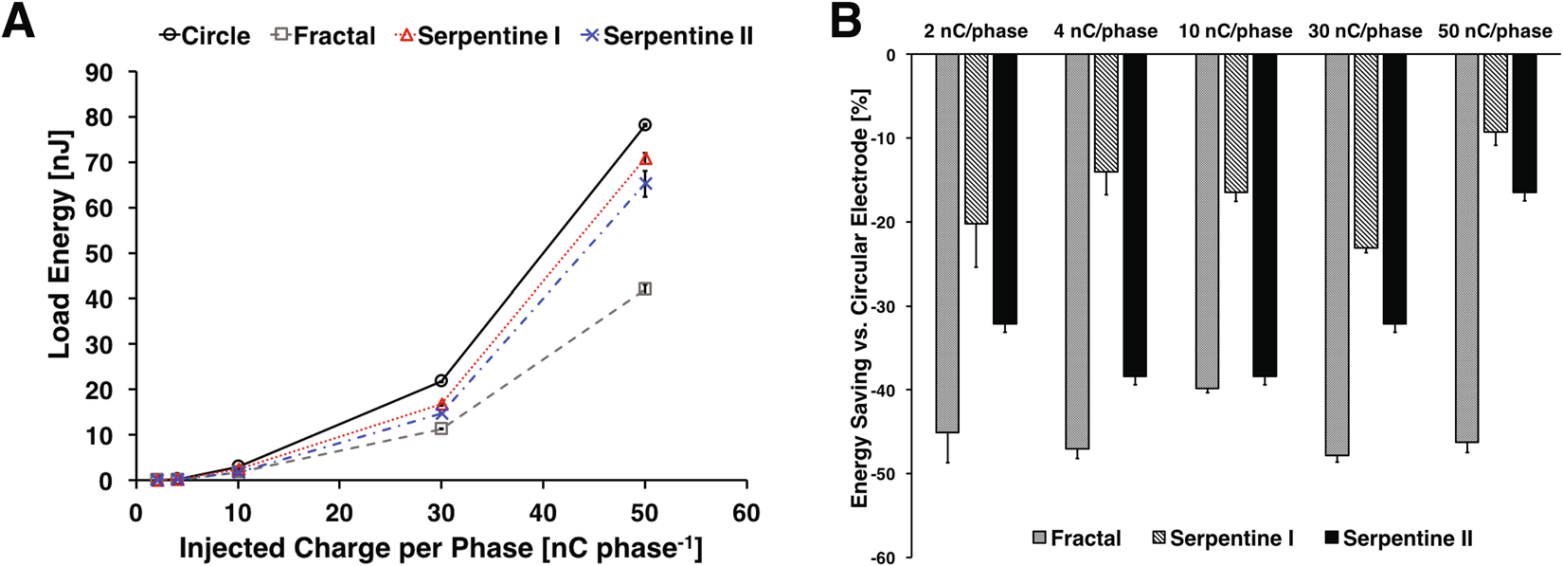
Comparison of load energy of the microelectrodes. (**A**) Energy consumption for a single cathodal pulse from the microelectrodes with different shape when the constant charge per phase was injected. (**B**) Energy consumption compared to the circular shaped microelectrode. During constant current stimulation the energy required to deliver the same amount of charge was significantly lower for fractal electrodes than other high PSA or circular electrodes.

The energy required to apply a cathodal pulse is described by the equation below (Foutz *et al*. 2012):

2 $${E}_{load}={\int }_{0}^{PW}{I}_{stim}{V}_{load}\,dt$$

where E_*load*_ is the energy consumed in the electrode and the solution, I_*stim*_ is the current amplitude for the pulse, V_*load*_ is the load voltage, and PW is the pulse-width. A comparison of the energy needed to apply the various current amplitudes is shown in Fig. 7. The fractal electrode needed significantly less energy (up to 47%) to deliver any given charge compared to the circular electrode. The serpentine II electrodes required less energy as well (up to 39%) compared to the circular electrode. However, as the charge level increases, the relative energy savings for the high PSA electrodes against the circular design decreased to less than 20%.

## Discussion

Here we experimentally demonstrated that electrodes with Vicsek fractal geometry may be more electrochemically efficient in charge transfer during neurostimulation than conventional circular microelectrodes and other microelectrodes with higher PSA ratio. Contrary to our expectation, the two different electrode designs with the same PSA ratio demonstrated significantly different electrochemical performance, which suggests the possibility of tailoring optimum electrode designs for various neurostimulation applications. The enhanced charge capacity afforded by the fractal design may also translate into a more energy efficient neurostimulation system with improved functional lifetime.

The CV responses of the four different electrodes revealed a similar PSA effect that has previously been reported in literature^28^. The fractal electrodes, however, showed significantly larger CSC than PSA-matched serpentine I electrodes. Compared to circular electrodes with the same surface area, CSC_*c*_ for fractal electrode was 57% higher whereas serpentine I electrode with the same PSA only showed 25% increase. The CSC_*c*_ for fractal electrode was similar to that of serpentine II electrodes with perimeter that is 1.6 times larger.

The CSC_*c*_ increase in Pt microelectrodes can be compared to that of IrOx microelectrodes. Although the magnitude of CSC_*c*_ in IrOx electrodes are typically 10 times larger than that of Pt electrodes, the change in CSC_*c*_ due to increasing PSA from 74 to 255 was approximately 31%, which is close to 25% increase we observed between circular (PSA = 40) and serpentine I (PSA = 256)^28,47^. This suggests that fractal design in IrOx may also translate into an even larger improvement in CSC_*c*_ as we demonstrated in Pt microelectrodes.

The charge injection limit measured by the voltage transient response of microelectrodes showed similar superior performance of the fractal designs. When pulsed with a charge-balanced current-controlled biphasic stimulation waveform, the fractal electrodes exhibited lowest driving potential (V_*dr*_) than the other electrode designs. The negative potential excursion (E_*mc*_) was the lowest in fractal electrodes as well. When extrapolated for charge injection limit, the fractal electrodes were able to deliver 73% more charge than circular design (510 *μ*C⋅*cm*^−2^ vs. 295 *μ*C⋅*cm*^−2^, Fig. 6) without reaching the water hydrolysis limit. Interestingly, the total current injected estimated in our constant voltage COMSOL model was also approximately 55% larger, which is close to the charge injection limit improvement we demonstrated.

Other groups have postulated that the increase in charge injection capability of high PSA may be attributable to decrease in access resistance and increase in ionic flux in high PSA electrodes^23,28^. Indeed, the estimated access resistance (i.e., V_*dr*_–E_*mc*_) for fractal and other high PSA ratio electrodes remained lower than that of circular electrodes at various charge injection levels (Fig. 6, Supplementary Fig. 4). This is also supported by our COMSOL analysis. Although our simplified numerical analysis does not take into account the impact of diffusion, the fractal and other high-PSA electrodes showed a lower overall resistance than circular electrode (Fig. 2). The impedance measured in PBS also suggest the lower overall electrode resistance for non-Euclidean electrodes (Fig. 3), however, it is difficult to distinguish the contribution due to Faradaic processes. Our EIS data in ferri-ferrocyanide does provide clearer evidence to demonstrate superior ionic flux in fractal and other non-Euclidean, high-PSA microelectrodes (Fig. 4, Table 4). Moreover, the equivalent circuit model analysis showed that the fractal electrodes have significantly higher capacitance than other electrodes, which suggests that these non-Euclidean microelectrodes have greater charge injection capacbility due to lower access resistance, superior ion flux, and lager capacitance (Supplementary Fig. 2).

Compared to the conventional stimulation electrodes found in DBS or SCS, the microelectrodes have significantly higher overall interface impedance due to their smaller size. Thus, creating a more efficient electrode design is critical in ensuring longevity of neurostimulation devices with high-density microelectrode array. In a recent simulation, Watterson *et al*. highlighted enhanced potential penetration capability of fractal electrodes^34^. The authors suggested that larger electrode bounding perimeter and the additional double layer capacitance afforded by the vertical side-wall, can lead to additional charge transfer that results in greater potential penetration. However, our work demonstrates that even without the added side-wall, the fractal designs may have greater charge injection capability due to superior Faradaic and non-Faradaic electrochemical processes. Although several groups have already proposed the idea of using high PSA electrode to improve charge transfer efficiency of stimulating electrodes, this work provides the first evidence to suggest that even more superior electrode may be possible by optimizing the geometry.

A critical next step is to evaluate the impact of electrode design in terms of its mechanical stability. As shown in our FEM (Fig. 2), the fractal design resulted in higher current density than other electrodes. The Pt electrodes are known to suffer from dissolution that scale with the magnitude of current density^48,49^. With the increased current density, we expect the dissolution process for these high PSA electrodes to be accelerated as well. Therefore, further study on the effects of fractal design on electrode dissolution is warranted altough the concern for electrode integrity may be mitigated by utilizing non-Pt electrodes materials or via imbalanced stimulation waveforms^50^. Finally, it is essential to investigate whether a more energy efficient neural stimulation can actually be achieved *in vivo* using these fractal microelectrode designs to confirm our electrochemical results.

## Methods

### Electrode design

A description of the Pt electrode geometries is provided in Table 1. The geometries of non-Euclidian electrodes were designed to match the surface area from circular shaped microelectrode with a diameter of 100 *μ*m (7854 *μ*m^2^) (Fig. 1). In Vicsek fractal, the area (*A*_*n*_) and perimeter (*P*_*n*_) at iteration *n* can be described using *A*_*n*_ = *l*^2^⋅5^*n*^ and *P*_*n*_ = 5*P*_*n*−1_−8*l* with *l* as the length of the initial square. Based on the area of circle microelectrode (7854 *μ*m^2^) and the resolution limit for microfabrication, the side of the smallest square unit of the fractal (l) was set to be 7.93 *μ*m with *n* = 3. The fractal and serpentine I electrodes were designed to have the same surface area as well as the perimeter. The desired PSA in serpentine electrodes were achieved by adjusting the radius of curvature and the length of straight portions. Fractal shape and serpentine I shape had approximately 6.35 times longer perimeter than the circular electrodes while serpentine II electrodes had 10 times longer perimeter (Table 1).

### Electrode fabrication

Figure 1C illustrates the overall fabrication flow. Platinum microelectrode of varying PSA were fabricated on 500 nm film of silicon nitride layer by plasma enhanced chemical vapor deposition (Axic, Milpitas, CA, USA). A photoresist (AZ1518, MicroChem, Newton, MA, USA) was spincoated over the silicon nitride layer and patterned to define microelectrode designs with different shapes. Pt film (100-nm-thick) was deposited on to the photoresist using a titanium (10 nm) as an adhesion layer. The electrode arrays were created using lift-off process. A 1.5-*μ*m-thick layer of polyimide (PI-2545, HD Microsystems, Parlin, NJ) was spin-coated over the wafer and cured as the insulation layer. The microelectrodes, counter electrode, and contact pads were created by reactive ion etching (RIE) with 20 sccm O_2_ at 100 W in 50 mTorr for 10 min using photoresist (AZ9260, MicroChem, Newton, MA, USA) as the etch mask.

### Numerical modeling of current density distribution

The finite element model was implemented using the stationary electric current mode of COMSOL 5.2a (COMSOL Inc., USA). The electric current mode solved the charge conservation equation for calculating current density distribution across the internal boundaries below.

3 $$\nabla J+\frac{\partial \rho }{\partial t}=0$$

where *J* is the current density, and *ρ* is the charge density. The current density is governed by the equations below:

4 $$J=\sigma E=-\sigma \nabla V$$

5 $$\nabla \cdot J+\frac{\partial \rho }{\partial t}={\nabla }^{2}V=0$$

with the electrical potential *V*. The electric currents mode ignores any Faradaic reactions that occur on the electrode surface. The model includes microelectrode domain, extracellular boundary with cylindrical shape, and five hemi-sphere domains with radius from 200 *μ*m to 1200 *μ*m to estimate current density distribution and total delivered current around the electrode (Fig. 2). The conductivity of simulated domain was 0.2 S m^−1^ to matched the brain tissue conductivity. The electric potential of −0.6 V was applied to electrode surface to simulate the cathodic limit of water window. The calculation of impedance in frequency domain were made from 10 Hz to 100 kHz by applying 10 mV AC voltage perturbation to electrode surface. The cylindrical outer boundary was grounded at 0 V, and the model was meshed using tetrahedral mesh elements.

### Cyclic voltammetry and electrochemical impedance spectroscopy

CV and EIS were performed using a custom microelectrode packaging platform (Fig. 1D). CV was measured using a commercial potentiostat (SP-200, Bio-Logic.Inc, Seyssinet-Pariset, France) in a standard three-electrode configuration using KCl saturated Ag/AgCl (RE-1CP, ALS Co., Ltd, Tokyo, Japan), along with the working and counter electrodes on the microelectrode array. CV was performed in phosphate-buffered saline solution (PBS) having composition of KH_2_PO_4_ 1.1 mM, NaCl 155 mM, and Na _2_ HPO_4_.H_2_O 3 mM with pH 7.4 (ThermoFisher Scientific, Waltham, MA, USA). All CV were measured at sweep rate of 50 mV⋅s^−1^ between potential range of −0.65 V and 0.85 V versus Ag/AgCl reference electrode. EIS measurements were obtained using the same experimental setup. The perturbation potential was sinusoidal 10 mV excitation voltage with the frequency range from 10 to 100 kHz in PBS. To gauge the impact of mass transport, EIS measurements were repeated using 10 mM solution of analyte ($\text{Fe}{(\text{CN})}_{6}^{-3/-4}$) in 0.1 M KCl. AC voltage perturbation of 30 mV was applied at the working electrode using sinusoidal signal from 10^-2^ Hz to 100 kHz at the equilibrium voltage 0.22 V vs. Ag/AgCl sat. The EIS data were recorded at 7 points per decade.

### Voltage transients

The voltage transient measurements using a charge-balanced biphasic current-controlled waveform were performed with an analog stimulus isolator (AM 2200, AM Systems, Sequim, WA, USA). A bespoke MATLAB program (R2016a, Mathworks, Natick, MA, USA) was used to generate stimulating waveform with specific pulse width, amplitude, and frequency. The pulses were injected into the electrode-electrolyte test cell, and a data acquisition board (NI USB-6353, National Instruments, Austin, TX, USA) was used to record the voltage transient responses. The charge-balanced biphasic pulse used in the experiments were cathodic-first current pulse with 100 *μ*s duration followed by 100 *μ*s inter-phase delay. The stimulating frequency was set at 50 Hz. The maximum negative potential excursion (E_*mc*_) was esteimated to be the potential immediately after the end of the cathodic pulse (Fig. 5A). The time delay at which the current becomes zero was measured to be approxmiately 12 *μ*s, and E_*mc*_ was recorded at 12 *μ*s following the end of the cathodic current pulse. V_*dr*_ is the negative driving voltage which is maximum voltage to deliver the cathodic current pulse.

## Electronic supplementary material

Supplementary Information
