# Supplementary material for: Electrochemical Evaluations of Fractal Microelectrodes for Energy Efficient Neurostimulation
Source: Sci Rep. 2018 Mar 12;8:4375. doi: 10.1038/s41598-018-22545-w (PMC5847577; doi:10.1038/s41598-018-22545-w)
Supplement: Supplementary file 1 — Supplementary Information [file 41598_2018_22545_MOESM1_ESM.pdf]

Electrochemical Evaluations of Fractal Microelectrodes for Energy Efficient Neurostimulation

Hyunsu Park[1], Pavel Takmakov[2], and Hyowon Lee[1,*]

[1]Weldon School of Biomedical Engineering, Birck Nanotechnology Center, Center for Implantable Devices, Purdue University, West Lafayette, IN, USA
[2]Division of Biology, Chemistry and Materials Science; Office of Science and Engineering Laboratories, Center for Devices and Radiological Health, U.S. Food and Drug Administration, White Oak Federal Research Center, Silver Spring, MD, US
[*]Correspondence to hwlee@purdue.edu

ABSTRACT

Advancements in microfabrication has enabled manufacturing of microscopic neurostimulation electrodes with smaller footprint than ever possible. The smaller electrodes can potentially reduce tissue damage and allow better spatial resolution for neural stimulation. Although electrodes of any shape can easily be fabricated, substantial effort have been focused on identification and characterization of new materials and surface morphology for efficient charge injection, while maintaining simple circular or rectangular Euclidean electrode geometries. In this work we provide a systematic electrochemical evaluation of charge injection capacities of serpentine and fractal-shaped platinum microelectrodes and compare their performance with traditional circular microelectrodes. Our findings indicate that the increase in electrode perimeter leads to an increase in maximum charge injection capacity. Furthermore, we found that the electrode geometry can have even more significant impact on electrode performance than having a larger perimeter for a given surface area. The fractal-shaped microelectrodes, despite having smaller perimeter than other designs, demonstrated superior charge injection capacity. Our results suggest that electrode design can significantly affect both Faradaic and non-Faradaic electrochemical processes, which may be optimized to enable a more energy efficient design for neurostimulation.

Supplementary Information

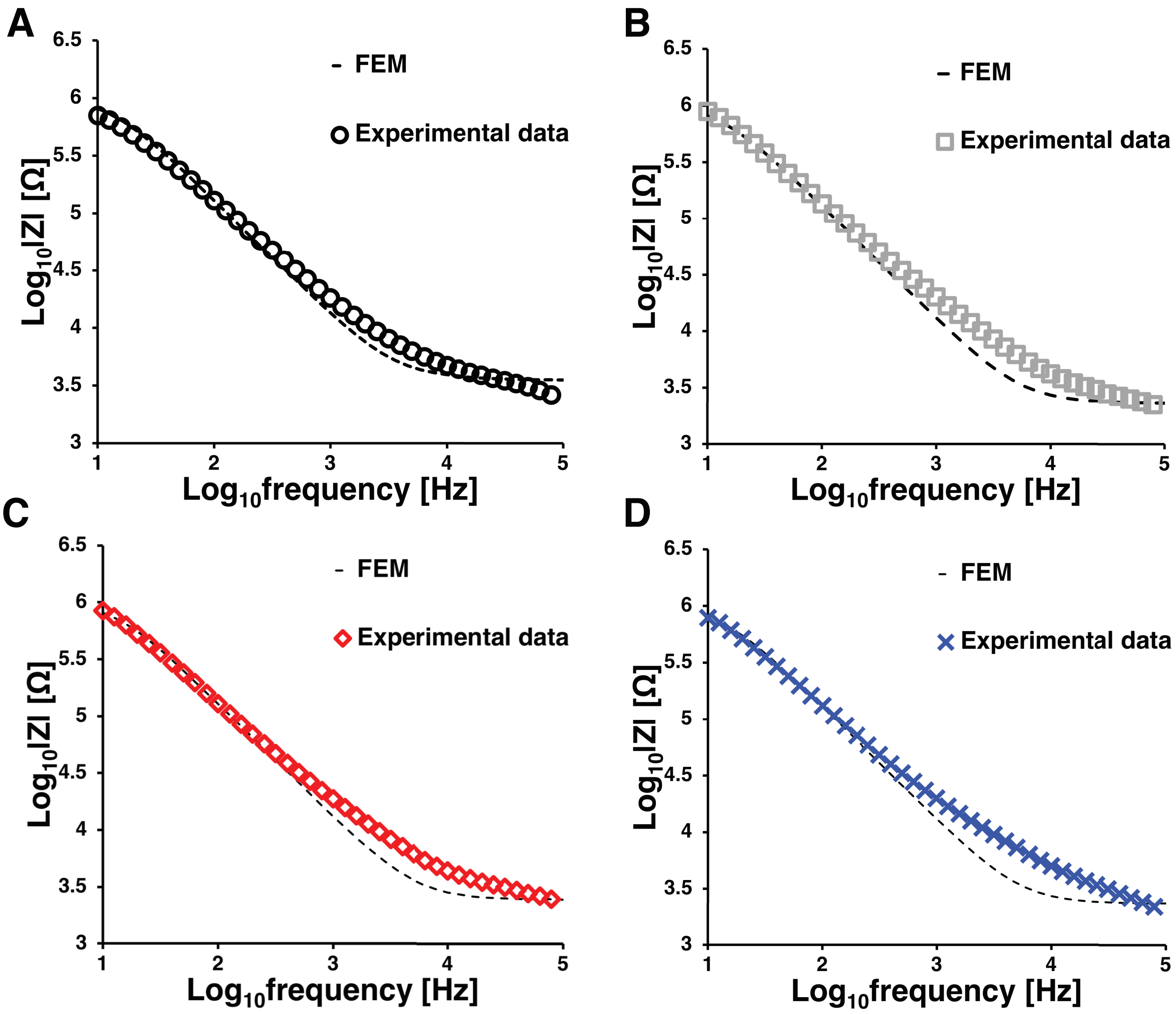

Figure 1. The impedance magnitudes simulated by FEM and EIS experimental data from (A) Circle, (B) Fractal, (C) Serpentine I, and (D) Serpentine II.

Frequency	Method	Circle	Fractal	Serpentine I	Serpentine II
10 Hz	Simulation	1.038×10^6	1.037×10^6	1.036×10^6	1.032×10^6
	Experiment	0.708×10^6	0.899×10^6	0.873×10^6	0.816×10^6
	Error [%]	31.80	13.23	15.71	20.92
1 kHz	Simulation	1.356×10^4	1.323×10^4	1.324×10^4	1.318×10^4
	Experiment	1.568×10^4	1.703×10^4	1.641×10^4	1.724×10^4
	Error [%]	15.58	28.68	23.87	30.79
100 kHz	Simulation	3540	2306	2454	2336
	Experiment	2402	2080	2267	1984
	Error [%]	32.13	9.781	7.609	15.06

Table 1. Comparison of the impedance from experiment and COMSOL simulation. (Unit:[Ω])

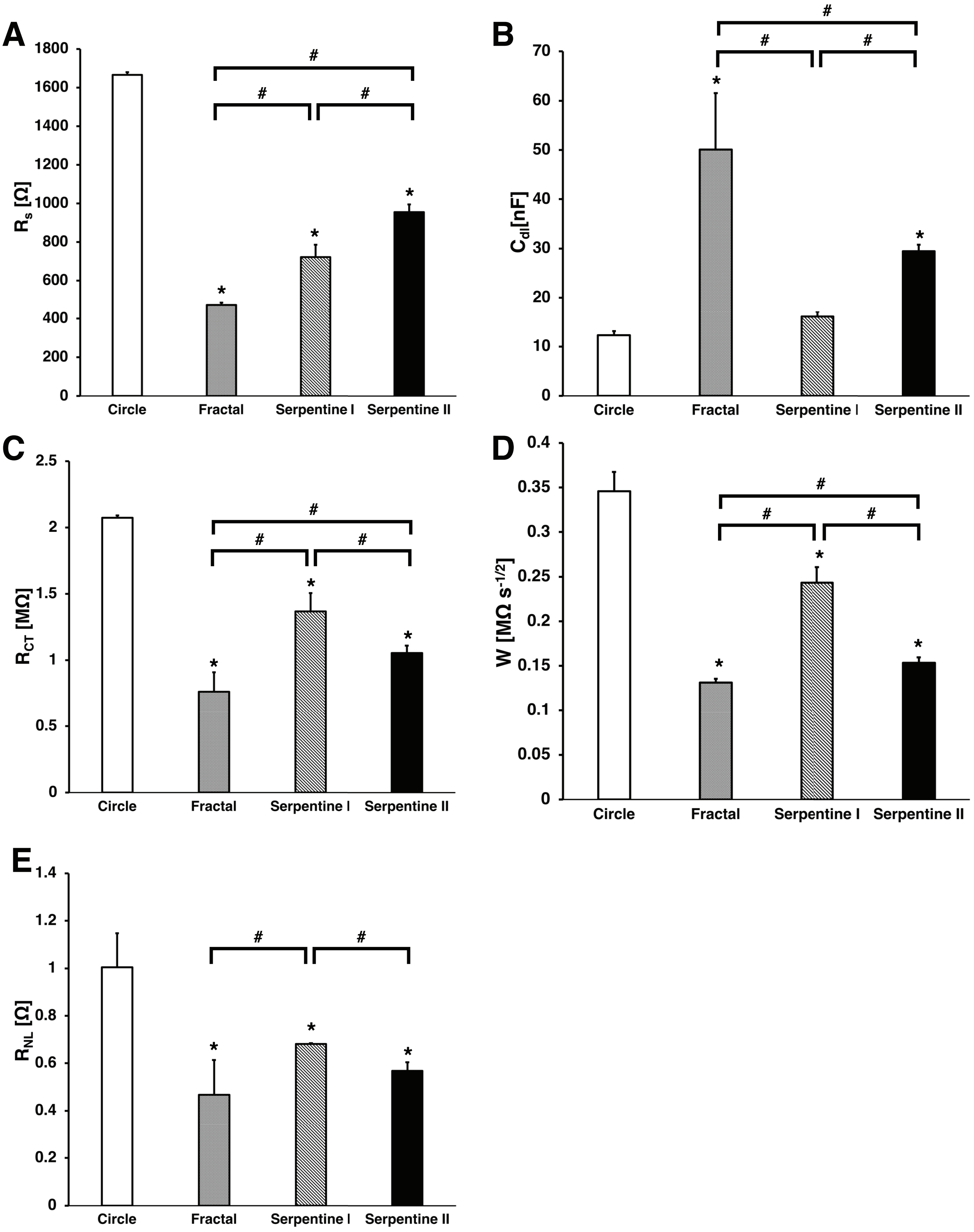

Figure 2. Fitted values (R_S, C_{dl}, R_{CT}, W, and R_{NL}) of each microelectrode (n=5 for each). ANOVA results revealed significant differences ($p<0.05$) as compared to circular electrodes ($*$), and significant differences ($p<0.05$) between fractal and serpentine I, serpentine I and serpentine II.

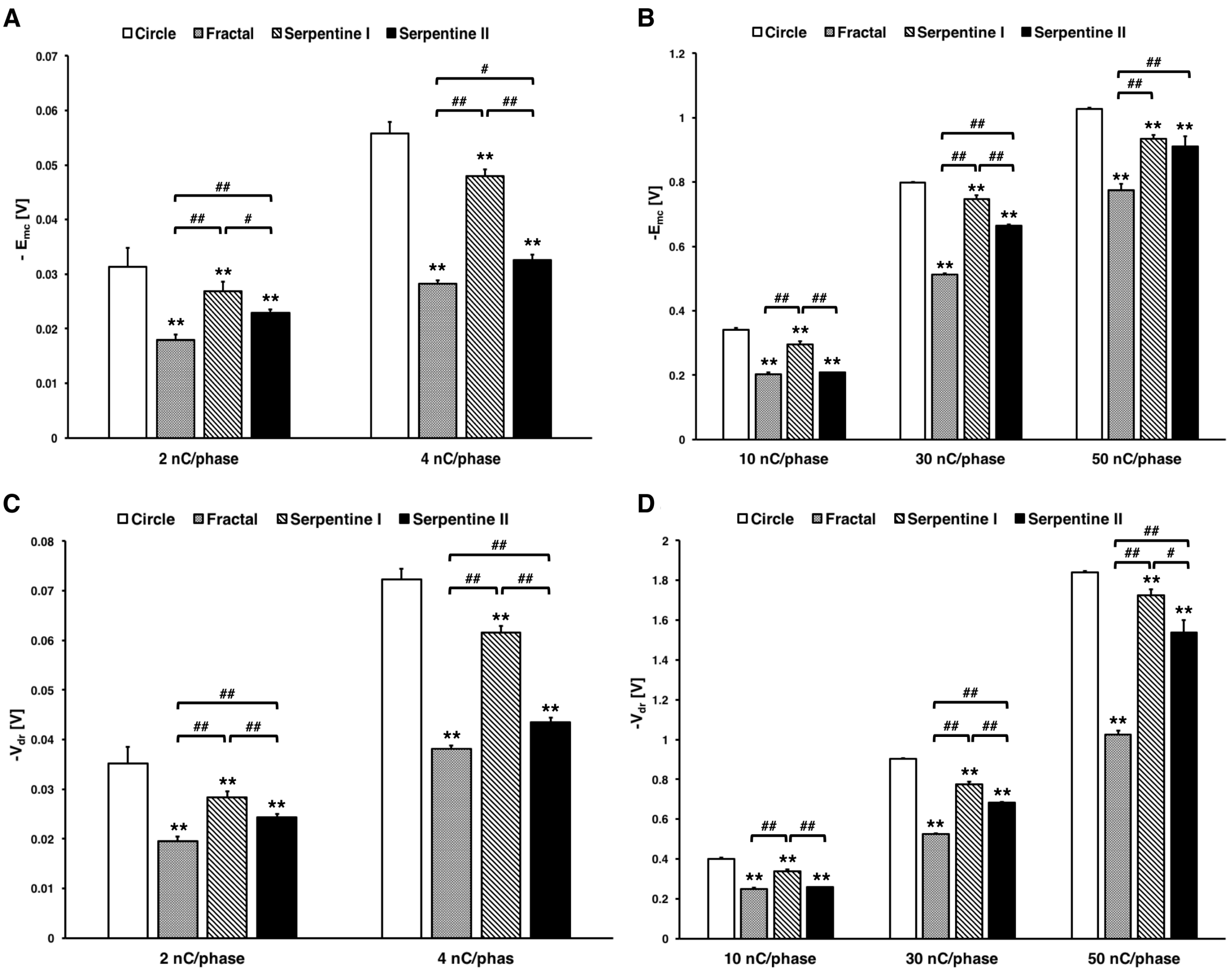

Figure 3. (A-B) Maximum negative potential excursion of the microelectrodes with different shapes. Post-hoc pairwise comparisons using Tukey's test (p<0.01). (C-D) Maximum driving voltage. Post-hoc pairwise comparison using Tukey's test (p<0.01).

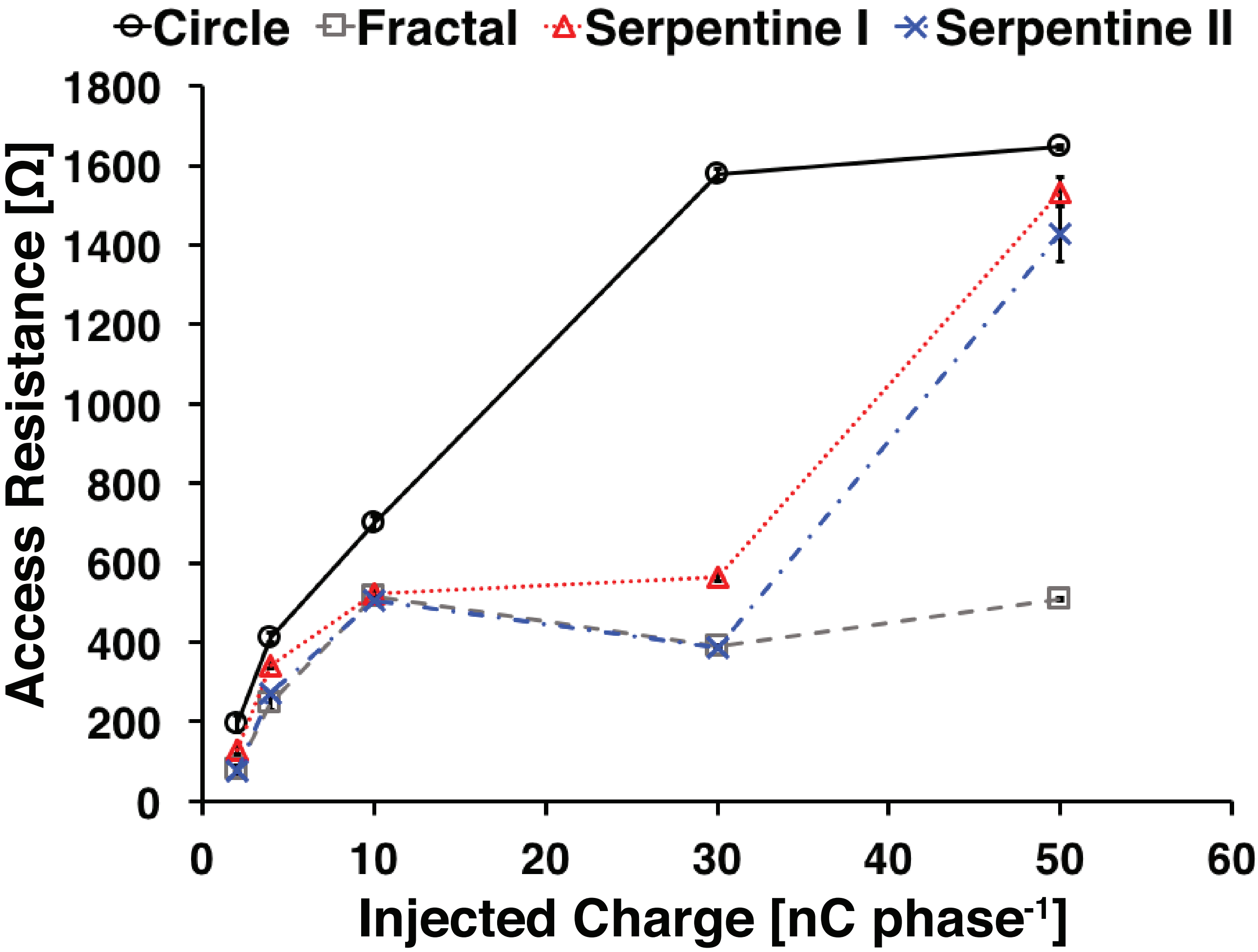

Figure 4. Estimated access resistance ($V_{dr} - E_{mc}$) at different injected charge levels for different electrodes.Note that the access resistance for fractal electrodes remain low for high charge levels which may explain their higher charge injection capacity.
